# Sevoflurane Postconditioning Attenuates Hypoxia/Reoxygenation Injury of Cardiomyocytes Under High Glucose by Regulating HIF-1α/MIF/AMPK Pathway

**DOI:** 10.3389/fphar.2020.624809

**Published:** 2021-02-22

**Authors:** Haiping Ma, Yongjie Li, Tianliang Hou, Jing Li, Long Yang, Hai Guo, Lili Li, Mingxiu Xin, Zhongcheng Gong

**Affiliations:** The First Affiliated Hospital of Xinjiang Medical University, Urumqi, China

**Keywords:** hypoxia inducible factor-1α, hypoxia/reoxygenation injury, sevoflurane post-conditioning, myocardial protection, diabetes

## Abstract

**Subject:** Cardiovascular disease, as a very common and serious coexisting disease in diabetic patients, and is one of the risk factors that seriously affect the prognosis and complications of surgical patients. Previous studies have shown that sevoflurane post-conditioning (SPostC) exerts a protective effect against myocardial ischemia/reperfusion injury by HIF-1α, but the protective effect is weakened or even disappeared under hyperglycemia. This study aims to explore whether regulating the HIF-1α/MIF/AMPK signaling pathway can restore the protective effect and reveal the mechanism of SPostC on cardiomyocyte hypoxia/reoxygenation injury under high glucose conditions.

**Methods:** H9c2 cardiomyocytes were cultured in normal and high-concentration glucose medium to establish a hypoxia/reoxygenation (H/R) injury model of cardiomyocytes. SPostC was performed with 2.4% sevoflurane for 15 min before reoxygenation. Cell damage was determined by measuring cell viability, lactate dehydrogenase activity, and apoptosis; Testing cell energy metabolism by detecting reactive oxygen species (ROS) generation, ATP content and mitochondrial membrane potential; Analysis of the change of HIF-1α, MIF and AMPKα mRNA expression by RT-PCR. Western blotting was used to examine the expression of HIF-1α, MIF, AMPKα and p-AMPKα proteins. HIF-1α and MIF inhibitors and agonists were administered 40 min before hypoxia.

**Results:** 1) SPostC exerts a protective effect by increasing cell viability, reducing LDH levels and cell apoptosis under low glucose (5 μM) after undergoing H/R injury; 2) High glucose concentration (35 μM) eliminated the cardioprotective effect of SPostC, which is manifested by a significantly decrease in the protein and mRNA expression level of the HIF-1α/MIF/AMPK signaling pathway, accompanied by decreased cell viability, increased LDH levels and apoptosis, increased ROS production, decreased ATP synthesis, and decreased mitochondrial membrane potential; 3. Under high glucose (35 μM), the expression levels of HIF-1α and MIF were up-regulated by using agonists, which can significantly increase the level of p-AMPKα protein, and the cardioprotective effect of SPostC was restored.

**Conclusion:** The signal pathway of HIF-1α/MIF/AMPK of H9c2 cardiomyocytes may be the key point of SPostC against H/R injure. The cardioprotective of SPostC could be restored by upregulating the protein expression of HIF-1α and MIF under hyperglycemia.

## Introduction

The incidence of diabetes is increasing year by year, and it is showing a trend of getting younger. It has become a global public health problem that seriously threatens human health. According to The International Diabetes Federation (IDF) in 2019, approximately 463 million adults worldwide suffered from diabetes, and it is estimated that the number of patients will exceed approximately seven million in the next 25 years (Saeedi et al., 2019). Studies have confirmed that long-term diabetes will produce many complications, including vascular endothelial dysfunction (Xu et al., 2019), exacerbating myocardial injure caused by ischemia/reperfusion (Ruan et al., 2019).

Cardiovascular disease, as the very common and serious coexisting disease in diabetic patients, is far more harmful than diabetes itself. Studies have found that the incidence of myocardial ischemia in diabetic patients is 1.45–2.99 times higher than that of non-diabetic patients (Aniskevich et al., 2017), and the incidence of perioperative heart-related complications is about 5 times higher than that of non-diabetic patients (Liu et al., 2016), which is a serious safety threat for surgical patients. Therefore, seeking effective myocardial protection strategies for diabetic patients during the perioperative period is an very important scientific issue.

Myocardial ischemia/reperfusion injury (I/RI) is a common clinical pathophysiological process, and it is also the main cause of perioperative cardiac adverse events and death (Rassaf et al., 2014). Sevoflurane is an inhaled anesthetic that is widely used in clinical and basic research. It has unique pharmacological characteristics such as stable induction and rapid recovery. Many studies have confirmed that sevoflurane post-conditioning (SPostC) can effectively alleviate I/RI of healthy cardiomyocytes (Pasqualin et al., 2016; Dong et al., 2019). However, hyperglycemia causes the myocardial protection of SPostC to disappear (Gao et al., 2016), and the specific mechanism is still unclear.

Hypoxia-inducible factor-1α (HIF-1α), as the initiating factor that regulates myocardial hypoxia and initiates the endogenous protective mechanism, is considered to be the main regulator of defense against hypoxic injury (Hasegawa et al., 2020). Our team’s previous studies confirmed that HIF-1α as a target to mediate the cardioprotective effect of SPostC (Yang et al., 2016), but the protein expression of HIF-1α is downregulated after the SpostC in diabetic myocardium, and the cardioprotective effect can be restored by upregulating the expression level of HIF-1α. However, the specific mechanism of HIF-1α-mediated myocardial protection of SPostC under high glucose is currently unclear. Macrophage migration inhibitory factor (MIF), as a downstream factor of HIF-1α, also plays an important role in the process of myocardial I/RI (Jankauskas et al., 2019; Voss et al., 2019). There is a close correlation between the expression levels of HIF-1α and MIF in myocardial tissue, and the expression of MIF increases with the up-regulation of HIF-1α (Pohl et al., 2017). MIF is also used as an upstream target protein to regulate adenylate activated protein kinase (AMPK) (Feng et al., 2018). AMPK is the central point of cell energy metabolism regulation and the main sensor for regulating energy state (Garcia and Shaw, 2017), could counter myocardial I/RI by promoting myocardial energy metabolism (Feng et al., 2018). Therefore, this study focuses on whether the HIF-1α/MIF/AMPK signaling pathway is a key point in mediating the protective effect of SPostC in hyperglycemia.

## Materials and Methods

### Cell Culture and Processing

The H9c2 rat embryonic cardiomyocyte cell line was obtained from Procell Life Science&Technology Co.,Ltd. China. The cell culture conditions consisted of DMEM (high glucose, 35 μM) medium + 10% FBS at 37°C, 5% CO_2_, and saturated humidity. H9c2 cells with good growth at 90% confluency were used to prepare a 5 × 10^4^ cell/mL single-cell suspension using complete medium. Cells were inoculated in 96-well plates and incubated for 24 h at 37°C in a 5% CO_2_ incubator. When the cells grew to 80% confluency, the supernatant was discarded, the adherent cells were washed twice with PBS, and serum-free DMEM (low glucose, 5 μM) medium was added.

### H/R Injury of Cardiomyocytes

Plates seeded with cardiomyocytes were placed in airtight, humidified and specifically modified chambers (Modular Incubator Chamber, MIC1-101, Billups-Rothenberg, United States) filled with 95% N_2_ and 5% CO_2_ to achieve an oxygen-deficient environment. Ventilation at 5 L/min for 15 min was used to achieve a 1% lower oxygen concentration in the chamber (the oxygen indicator card will change from blue to red when the oxygen concentration in the sealed chamber is less than 0.1%). Cells were incubated in 95% air and 5% CO_2_ at 37°C for 3 has reoxygenation. The N + CON group and H + CON group were kept in normoxic conditions for 3 h at appropriate times, while cells of other groups were exposed to hypoxia.

### Sevoflurane Post-conditioning of Cardiomyocytes

According to the previous study (Yu et al., 2017), a Vapor 2000 sevoflurane vaporizer (Drager, Lubeck, Germany) was used to apply a gas mixture containing 97.6% O_2_ and 2.4% sevoflurane. Briefly, an in-line sevoflurane vaporizer fed a supply of gas mixture containing 97.6% O_2_ and 2.4% sevoflurane for at least 10 min until the desired sevoflurane concentration (2.4%) was achieved. Concentrations of sevoflurane and O_2_ were monitored using an anesthetic analyzer (Drager Famous, Lubeck, Germany) in the outlet. The gas flow rate was 2 L/min. After the cells were treated for 15 min, they were taken out immediately and incubated in a 5% CO_2_ cell incubator for 165 min at 37°C. Other groups that do not require the intervention of sevoflurane received inhalation of pure oxygen (100% oxygen) for the same time interval.

### Pharmacological Inhibitor and Agonist

Cardiomyocytes were incubated with the HIF-1α inhibitor YC-1 (Selleck, United States) for 40 min at 100 μM before hypoxia, MIF inhibitor ISO-1 (Selleck, United States) for 40 min at 5 μM before hypoxia; HIF-1α agonist DMOG (Selleck, United States) for 40 min at 1 mM before hypoxia, MIF agonist MIF20 (Shenggong, China) for 40 min at 100 ng/ml before hypoxia. Investigate their modulating effects on following SpostC under high glucose conditions.

### Experimental Grouping

**FIGURE 1:**
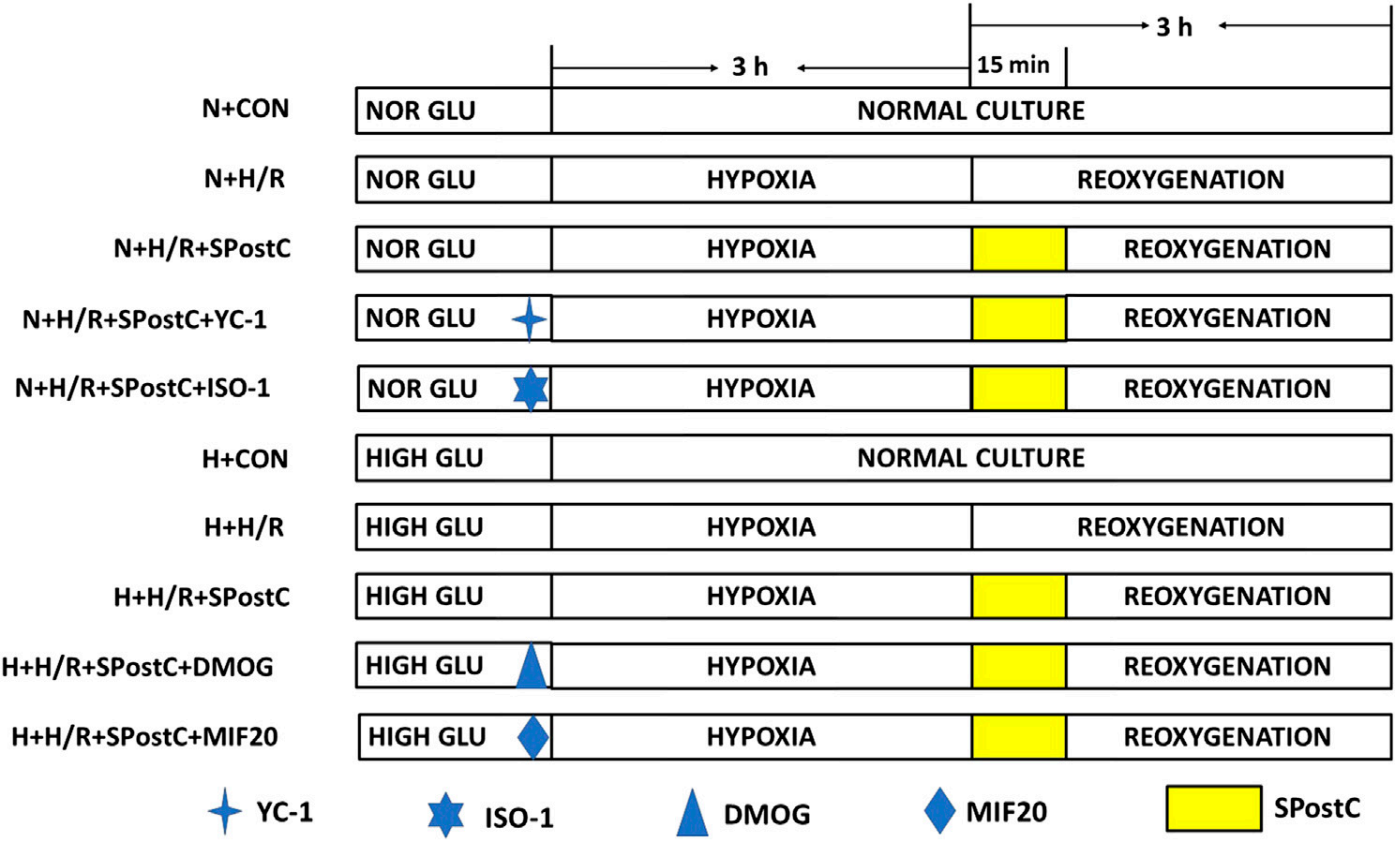
Experimental groups with respective protocols. H9c2 cardiomyocytes were randomly divided into N + CON, N + H/R, N + H/R + SPostC, N + H/R + SPostC + YC-1, N + H/R + SPostC + ISO-1, H + CON, H + H/R, H + H/R + SPostC, H + H/R + SPostC + DMOG and H + H/R + SPostC + MIF20 groups.

Cardiomyocytes incubated for 24 h were randomly divided into five groups (Figure 1):

Control (N + CON) group: H9c2 cells were continuously cultured in DMEM low-glucose medium (5 mM) containing 10% FBS without any interventions for 6 h.

Hypoxia/reoxygenation (N + H/R) group: H9c2 cells were placed in an airtight container with 95% N_2_ and 5% CO_2_ for 3 h, then reoxygenation (95% air and 5% CO_2_) with the addition of fresh low glucose DMEM with 10% FBS at 37°C, for a total 3 h of reoxygenation.

SPostC (N + H/R + SPostC) group: H9c2 cells were exposed to 2.4% sevoflurane for 15 min at the beginning of reoxygenation after 3 h of hypoxia, and then incubated in a 5% CO_2_ cell incubator for 165 min.

HIF-1α inhibitor (N + H/R + SPostC + YC-1) group: YC-1 (10 μM) was added to the medium for 40 min before hypoxia, followed by H/R injury and SpostC (same as N + H/R + SPostC group).

MIF inhibitor (N + H/R + SPostC + ISO-1) group: ISO-1 (5 μM) was added to the medium for 40 min before hypoxia, followed by H/R injury and SPostC (same as the N + H/R + SPostC group).

H + H/R group: H9c2 cardiomyocytes at baseline were incubated in high-glucose medium for 48 h followed by H/R (same as the N + H/R group).

H + H/R + SPostC group: Cardiomyocytes at baseline were incubated in a high-glucose medium for 48 h, followed by H/R and SPostC (same as N + H/R + SPostC group).

HIF-1α agonist (H + H/R + SPostC + DMOG) group: DMOG (1 mM) was added to the medium for 40 min before hypoxia, followed by H/R injury and SPostC (same as H + H/R + SPostC group).

MIF agonist (H + H/R + SPostC + MIF20) group: MIF20 (100 ng/ml) was added to the medium for 40 min before hypoxia, followed by H/R injury and SPostC (same as H + H/R + SPostC group).

### Detection Methods

#### Determination of Cell Viability

Cell viability was determined by the CCK-8 method. A total of 100 μL of 10% CCK-8 solution was added to each well of a 96-well plate, which was then incubated at 37°C. After 2 h, the optical density values were measured at a wavelength of 450 nm using a plate reader.

#### Determination of Lactate Dehydrogenase Content

According to the instructions of the LDH cytotoxicity test kit, cell supernatants of each well of the 96-well plate were collected and transferred to a new 96-well plate in each experimental group, and 60 μL of LDH test solution was added to each well. The plate was wrapped with foil and placed on a shaker to incubate for 30 min at room temperature. The absorbance values were then measured at 490 nm. The absorbance value was proportional to the LDH content.

#### Flow Cytometry Analysis of Cardiomyocyte Apoptosis

Cell apoptosis was assessed by PE Annexin V Apoptosis Detection Kit I (BD Biosciences, United States) according to the manufacturer’s protocol. The whole operation process was protected from light. In brief, cells washed with ice-cold phosphate-buffered saline (PBS) were resuspended in binding buffer. The solution (1 × 10^5^cells) supplemented with PE Annexin V and 7-AAD incubated for 15 min at room temperature. The apoptotic cells were identified by flow cytometry (Beckman Coulter, United States).

#### Reactive Oxygen Species Detection

hiPS-CMs were incubated with 2′,7′-dichlorofluorescein diacetate (H2DCF-DA, Beyotime) (10 µmol/L) for 30 min at 37°C in dark and then rinsed with PBS three times. The fluorescence was observed with a confocal laser microscope at 488 nm excitation and 525 nm emission wavelengths.

#### ATP Content Measurement

In brief, an ATP assay kit was used to quantify myocardial ATP based on the luciferin-luciferase reaction. The concentration of myocardial phosphocreatine was measured via reverse-phase high-performance liquid chromatography. A glycogen detection kit was used to determine the concentration of glycogen in the myocardium.

#### Mitochondrial Membrane Potential Measurement

To evaluate the redistribution of mitochondrial membrane potential (Δψm), JC-1 (Millipore, United States) was used to incubate cardiomyocytes following the manufacturer’s instructions. Briefly, Cells were incubated with 10 nmol/L JC-1 staining solution at 37°C free light for 10 min. The images were captured using a fluorescence inverted microscope (LEICA-DMI4000B, Germany). Red and green fluorescence intensities were analyzed respectively using Image J software, and the ratio of the red: green fluorescence was proportional to the Δψm. Thirty randomly chosen cardiomyocytes per treatment group were analyzed (n = 3 independent experiments with 10 incubated cardiomyocytes per experiment).

#### RT-PCR Detection

The primer sequence is synthesized with β-actin as an internal reference.

**Table:** 

Name of primers	Sequences of primers	Product length
HIF-1α forward	5′-GCA​ACT​GCC​ACC​ACT​GAT​GA-3′	152 bp
HIF-1α reverse	5′-GCT​GTC​CGA​CTG​TGA​GTA​CC-3′	
MIF forward	5′-GGA​CCG​GGT​CTA​CAT​CAA​CT-3′	115 bp
MIF reverse	5′-CAA​GAC​TCG​AAG​AAC​AGC​GG-3′	
AMPKα forward	5′-GAT​CCA​AGA​GCC​GAG​TTG​CT-3′	136 bp
AMPKα reverse	5′-TCC​GTT​CTA​TGC​GCT​GGA​TT-3′	
Β-Actin forward	5′-CCC​ATC​TAT​GAG​GGT​TAC​GC-3′	150 bp
Β-Actin reverse	5′-TTT​AAT​GTC​ACG​CAC​GAT​TTC-3′	

The nucleus pulposus tissue was ground and added to thelysate. TRIzol reagent was used to extract total RNA. After extraction, the concentration and purity of total RNA were identified using 1% agarose gel and UV spectrophotometer. Reverse transcription of total RNA was performed according to the cDNA kit instructions. A portion of the cDNA product was subjected to subsequent experiments, and the excess product was stored at −20°C until use. ABI Prism 7900 PCR instrument was used for PCR amplification, PCR system: PCR mix (2XTamix) 12.5 L, DNA template 2.0 L, 1.0 L each of upstream and downstream primers, and double distilled water to make up to 25 L. PCR reaction conditions: 95°C for 5 min, 95°C for 30 s, 60°C for 30 s, 72°C for 1 min. A total of 40 amplifications were performed at this time, and the final extension was at 72°C for 5 min β-actin was used as an internal reference and the experiment was conducted 3 times in total.

#### Western Blotting

The treated cells were collected and lyzed in RIPA buffer (Thermo Fisher Scientific, United States) supplemented with phosphatase and protease inhibitors (Thermo Fisher Scientific, United States). The extracted proteins were separated by SDS-PAGE and then transferred to PVDF membrane (Millipore Co., Bedford, MA). Proteins were probed with primary antibodies (1:1,000) including rabbit antibodies MIF, mouse antibody HIF-1α (all from Abcam, United States), rabbit antibodies AMPKα, phospho-AMPK(Thr172) (Cell Signaling Technology, United States), beta-Actin Loading Control antibody Mouse MAb (BioEngX, China), followed by incubation with corresponding secondary antibody conjugated with alkaline phosphatase (goat antirabbit IgG or goat anti-mouse IgG (all from Abcam, United States)). An alkaline phosphatase chromogen (BCIP/NBT, Invitrogen, United States) was used to detect bound antibodies. Signals were detected and quantified with Image Lab 4.0 software (Bio-Rad Laboratories, United States).

#### Statistical Analysis

Statistical analysis was performed using the GraphPad Prism 6.0. The data are presented as the mean ± SD. One-way analysis of variance (ANOVA) was used for statistical analyses of data obtained within the same group and between groups, respectively, followed by Tukey’s test for multiple comparisons of group means. *p* < 0.05 was considered statistically significant.

## Results

### Cardiomyocytes are Damaged Under High Glucose Condition and the Cardioprotective Effect Mediated by SpostC is Weakened

**FIGURE 2:**
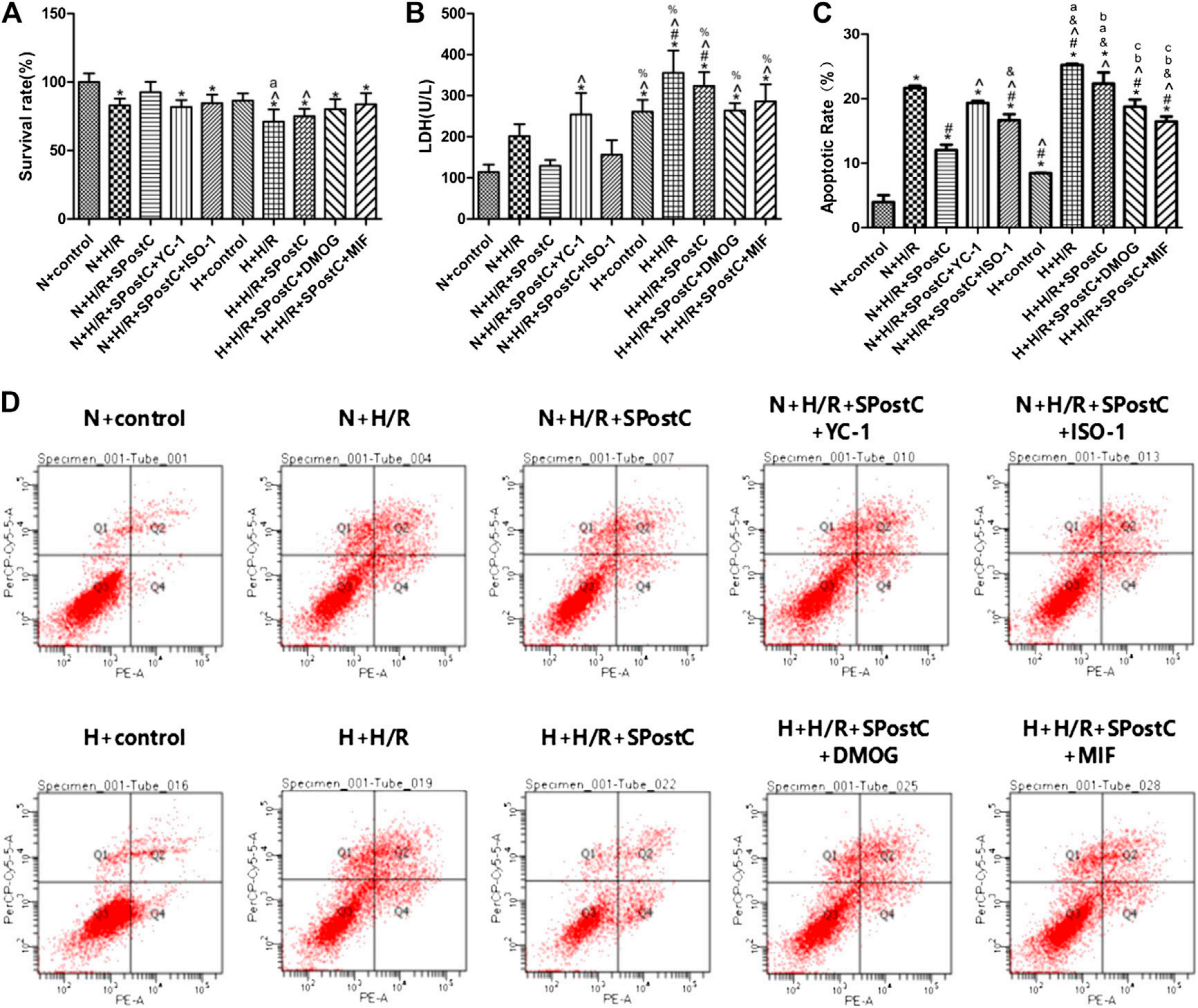
High glucose concentration was cell-damaging and abolished SPostC-induced cardioprotection. **(A)** Cell viability: Compared with N + H/R group, N + H/R + SPostC group improved cell viability, but H + H/R + SPostC group failed to increase cell viability (n = 5/group). **(B)** LDH activity: Compared to N + H/R group, N + H/R + SPostC group significantly reduced LDH content, but H + H/R + SPostC group failed to reduce LDH content (n = 3/group). **(C,D)** Flow cytometry to measure apoptosis: Compared with N + H/R group, N + H/R + SPostC group reduced cell apoptosis, but H + H/R + SPostC group failed to effectively reduce cell apoptosis (n = 3/group). Data represent mean ± SD, vs. N + CON group ^*^ *p* < 0.05; vs. N + H/R group ^#^ *p* < 0.05; vs. N + H/R + SPostC group ^^^ *p* < 0.05; vs. N + H/R + SPostC + YC-1 group ^&^ *p* < 0.05; vs. N + H/R + SPostC + ISO-1 group ^%^ *p* < 0.05; vs. H + CON group ^a^ *P* < 0.05; vs. H + H/R group ^b^ *P* < 0.05; vs. H + H/R + SPostC group ^c^ *P* < 0.05.

Cell viability, LDH activity, and apoptosis reflect the extent of cell damage. In this study, compared with N + H/R group, N + H/R + SPostC group significantly reduces cell damage, which is shown as increased cell viability (Figure 2A), and decreased LDH content (Figure 2B); Simultaneously accompanied by reduced the rate of cell apoptosis, which is detected by flow cytometry (Figures 2C,D); However, the damage of myocardial cells was increased again when the HIF-1α inhibitor (YC-1) and MIF inhibitor (ISO-1)were used respectively (Figures 2A–C). In the high glucose state, the cell viability of all groups were lower than normal culture group (Figure 2A). The LDH test in the high glucose state showed that all groups were higher than the normal culture group (Figure 2B). Similarly, the apoptosis of all groups under high glucose condition was more serious than normal culture group (Figures 2C,D). After HIF-1α agonist (DMOG, 1 mM) and MIF agonist (MIF20, 100 ng/ml) were used respectively in H + H/R + SPostC group, the myocardial protective effect of SPostC was restored. The results showed that SPostC attenuated the H/R damage of cardiomyocytes under high glucose conditions, but the protective effect of SPostC was reversed by activating HIF and MIF.

### The Effect of SPostC on the mRNA Expression of HIF-1α, MIF and AMPKα in the H/R Injury of Cardiomyocytes Under Low Glucose and High Glucose Conditions

**FIGURE 3:**
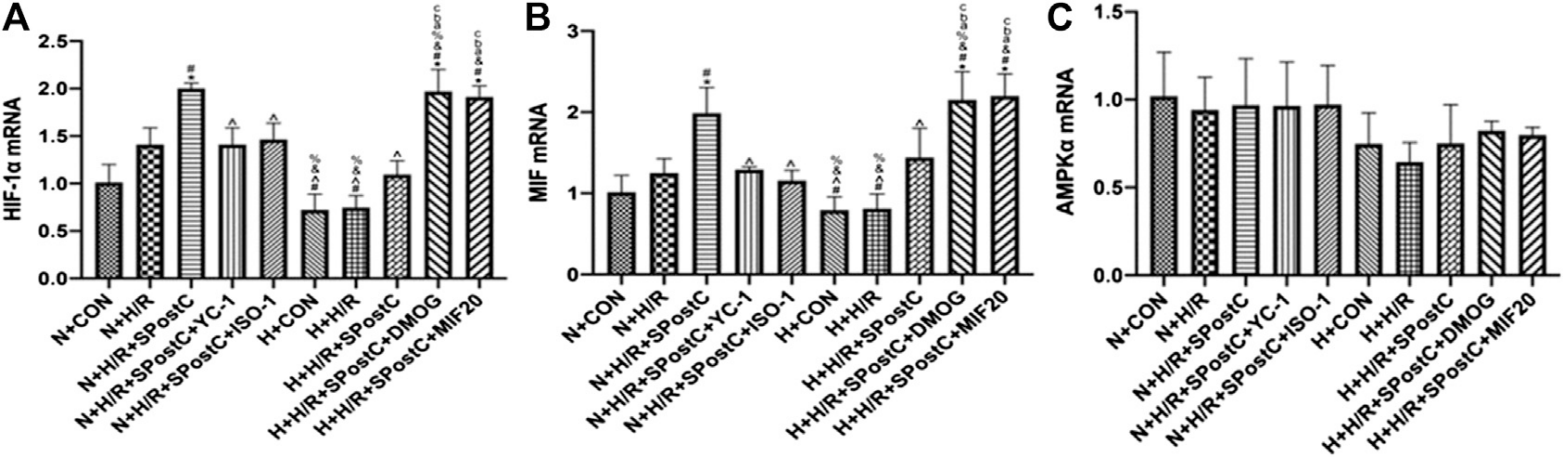
The effect of SPostC on the mRNA expression of HIF-1α, MIF and AMPKα in the H/R injury of cardiomyocytes under low glucose and high glucose conditions. **(A)** The mRNA expression of HIF-1α. **(B)** The mRNA expression of MIF. **(C)** The mRNA expression of AMPKα. Data represent mean ± SD, vs. N + CON group *p < 0.05; vs. N + H/R group ^#^ *p* < 0.05; vs. N + H/R + SPostC group ^^^ *p* < 0.05; vs. N + H/R + SPostC + YC-1 group ^&^ *p* < 0.05; vs. N + H/R + SPostC + ISO-1 group %*p* < 0.05; vs. H + CON group ^a^ *P* < 0.05; vs. H + H/R group ^b^ *P* < 0.05; vs. H + H/R + SPostC group ^c^ *P* < 0.05.

The mRNA expression of HIF-1α and MIF increased after H/R injury in cardiomyocytes. Compared with the N + H/R group, SPostC significantly increased the mRNA expression of HIF-1α and MIF. However, after using HIF-1α inhibitor YC-1 and MIF inhibitor ISO-1, the mRNA expression of HIF-1α and MIF decreased. In addition, under the condition of high glucose, compared with N + H/R + SPostC group, H + H/R + SPostC group did not significantly increase the mRNA expression of HIF-1α and MIF. It is confirmed that at the level of mRNA expression, the cardioprotective effect of SPostC is weakened under the condition of high glucose. But after the use of HIF-1α agonist DMOG and MIF agonist MIF20, the mRNA expression of HIF-1α and MIF increased significantly. We found that there was no difference in the expression of AMPKα mRNA among all groups, and it was concluded that there was no significant difference in the expression of AMPKα at mRNA level. Our results confirmed that at the level of mRNA expression, SPostC mediates HIF-1α/MIF/AMPK signal transduction to play a cardioprotective effect under low glucose conditions. Under the condition of high glucose, the signal transduction of HIF-1α/MIF/AMPK is weakened, which leads to the weakening of the protective effect of SPostC (Figure 3).

**FIGURE 4:**
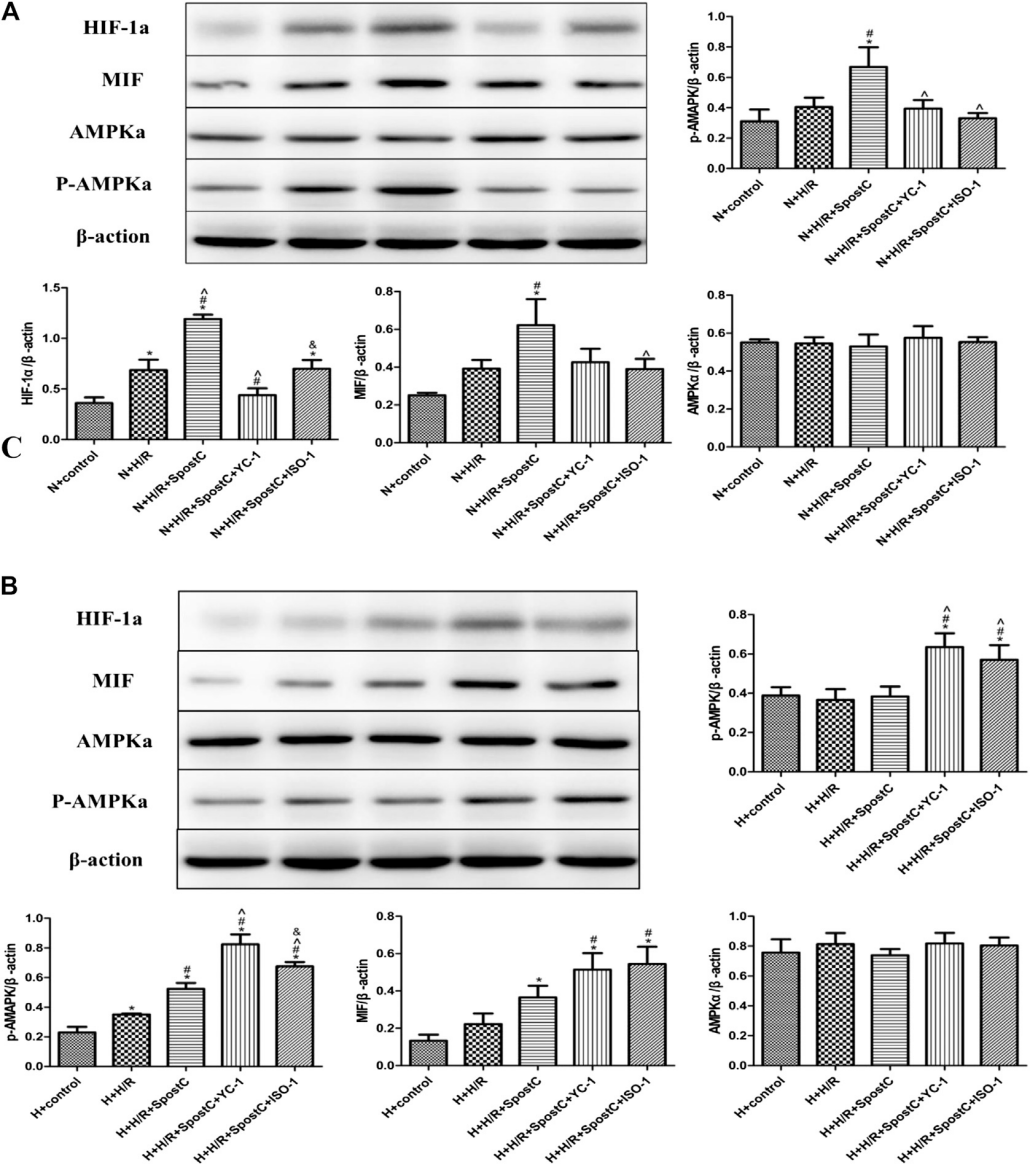
SPostC exerts its cardioprotective effect by HIF-1α/MIF/AMPK signal pathway under low glucose condition, but the function is inactivated under high glucose condition. **(A)** Western blot images of each group under normal glucose condition; **(B)** Western blot images of each group under high glucose condition; Under normal culture, SPostC significantly upregulated HIF-1a, MIF and p-AMPKα protein expression. After YC-1 and IOS-1 administration, the expression of HIF-1a MIF and p-AMPKα was downregulated. Data represent mean ± SD, vs. N + CON group *p < 0.05; vs. N + H/R group ^#^ *p* < 0.05; vs. N + H/R + SPostC group ^^^ *p* < 0.05; vs. N + H/R + SPostC + YC-1 group ^&^ *p* < 0.05; vs. H + control group ^a^ *P* < 0.05; vs. H + H/R group ^b^ *P* < 0.05; vs. H + H/R + SPostC group ^c^ *P* < 0.05.

### SPostC Exerts Cardioprotective Effect by HIF-1α/MIF/AMPK Signal Pathway Under Low Glucose Condition, but the Function is Inactivated Under High Glucose Condition

Western blot was used to detect the protein expression levels of HIF-1α, MIF, AMPKα and p-AMPKα in each group of cells (Figure 4). In this study, under the condition of low glucose (5 μM), compared with the N + CON group, H/R injury of cardiomyocytes slightly increasedthe protein expression levels of HIF-1α, MIF, p-AMPKα; compared with the N + H/R group, SPostC increased HIF-1α, MIF, and p-AMPKα protein expression level; Compared with N + H/R + SPostC group, HIF-1α inhibitor YC-1 and MIF inhibitor IOS-1 inhibited HIF-1α, MIF, and p-AMPKα protein expression levels. In the high glucose state (35 μM), compared with the H + CON group, the HIF-1α, MIF and p-AMPKα protein levels in the H + H/R group were higer; compared with the H + H/R group, the HIF-1α, MIF and p-AMPKα protein levels were no significant difference expression in the H + H/R + SPostC group; compared with H + H/R + SPostC group, HIF-1α agonist DMOG and MIF agonist MIF20 upregulated the protein expression levels of HIF-1α, MIF and p-AMPKα. We found that the protein expression of AMPKα had no obvious change trend, which was consistent with the expression level of AMPKα mRNA above. Therefore, we believe that at the protein level, the HIF/MIF signal axis regulates the phosphorylation of AMPKMP Therefore, our results confirm that SPostC-mediated HIF-1α/MIF/AMPK signal transduction plays a cardioprotective role in cardiomyocytes under low glucose conditions. Under the condition of high glucose, HIF-1α/MIF/AMPK signal transduction is weakened, which weakens the protective effect of SPostC, but the myocardial protective effect of SPostC is restored after exogenous up-regulation of HIF-1 α and MIF expression.

### Regulation of HIF-1α/MIF/AMPK Signal Transduction to Restore the Myocardial Protective Effect of SpostC Under High Glucose

**FIGURE 5:**
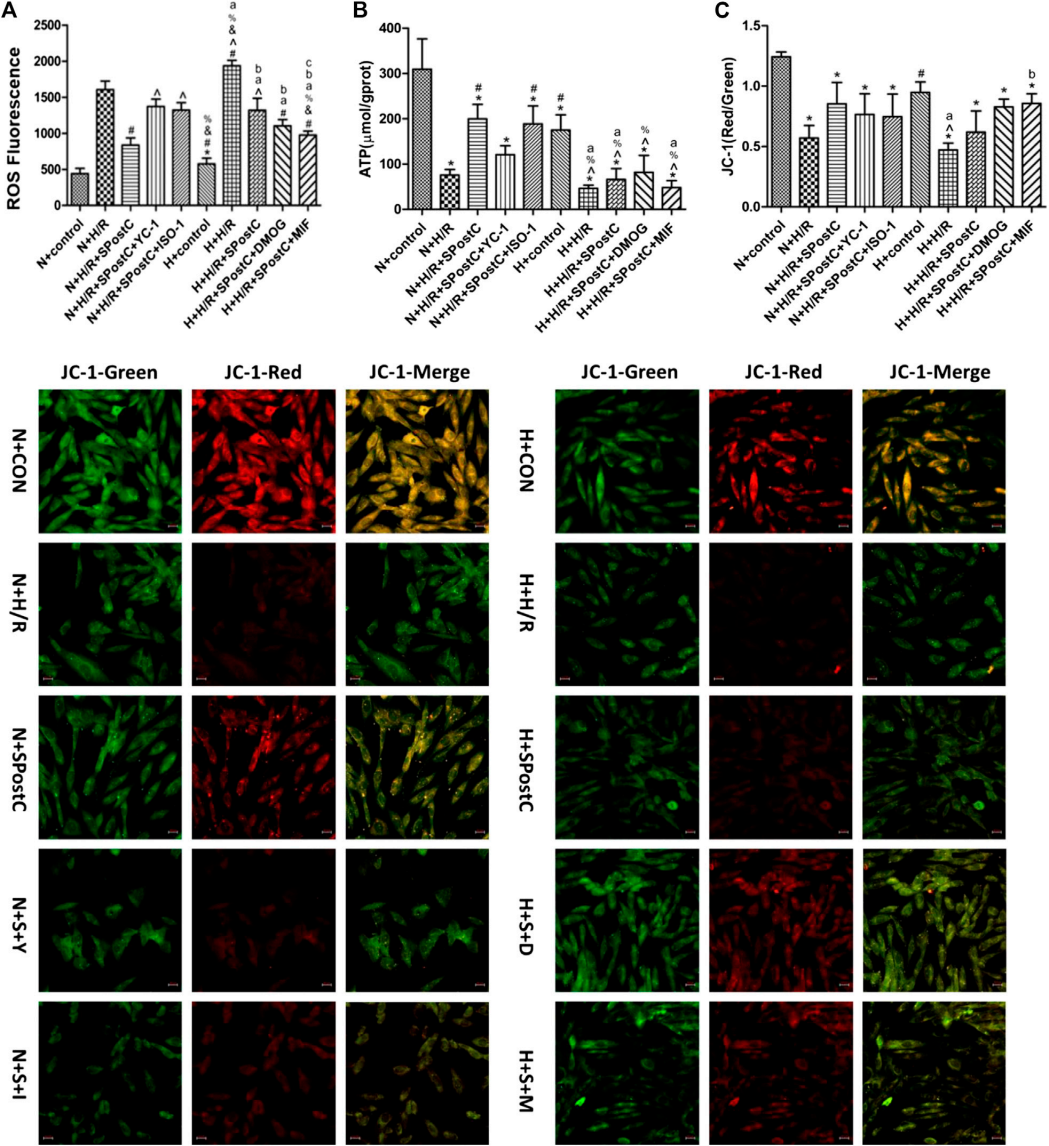
Regulate HIF-1α/MIF/AMPK signaling pathway restored SPostC-induced cardioprotective effects in a high glucose condition. **(A)** ROS synthesis: Compared with N + H/R group, N + H/R + SPostC group reduced ROS synthesis, but H + H/R + SPostC group failed to reduce ROS synthesis; Compared with N + H/R + SPostC group, YC-1 group and IOS-1 group promoted ROS synthesis; Compared with H + H/R + SPostC group, DMOG group and MIF20 group reduced ROS synthesis (n = 3/group). **(B)** ATP content: Compared with N + H/R group, N + H/R + SPostC group significantly increased ATP content, but H + H/R + SPostC group ATP content decreased; compared with N + H/R + SPostC group, YC-1 group and IOS-1 group decreased ATP content; compared with H + H/R + SPostC group Compared with DMOG group and MIF20 group, ATP content increased (n = 3/group). **(C)** Representative images of JC-1 were capture using a fluorescence inverted microscope in cardiomyocytes (200×). The results showed that HIF-1α agonist DMOG and MIF agonist MIF20 increased the ratio of red to green fluorescence intensity (n = 3/group). Data represent mean ± SD, vs. N + CON group *p < 0.05; vs. N + H/R group #*p* < 0.05; vs. N + H/R + SPostC group ^*p* < 0.05; vs. N + H/R + SPostC + YC-1 group & *p* < 0.05; vs. N + H/R + SPostC + ISO-1 group %*p* < 0.05; vs. H + control group ^a^ *P* < 0.05; vs. H + H/R group ^b^ *P* < 0.05; vs. H + H/R + SPostC group ^c^ *P* < 0.05.

Experiments to detect cell ROS production, ATP content and the detection of mitochondrial membrane potential reflect that the upregulated of HIF-1α/MIF/AMPK signaling pathway restored SPostC-induced cardioprotective effects in a high glucose condition. The results showed that the generation of ROS in the N + H/R group and H + H/R group was higher than that in the N + CON group and H + CON group; but the N + H/R + SPostC group significantly reduced ROS generation; the HIF-1α inhibitor YC-1 group and the MIF inhibitor IOS-1 group promoted ROS generation, but the HIF-1α agonist DMOG group and the MIF agonist MIF20 group significantly reduced ROS generation (Figure 5A). ATP content detection showed that the ATP content of N + H/R group and H + H/R group was significantly lower than that of N + CON group and H + CON group; the ATP content of N + H/R + SPostC group and H + H/R + SPostC group was significantly higher than that of N + H/R group and H + H/R group; compared with the H + H/R + SPostC group, the ATP content of the YC-1 group and the IOS-1 group decreased slightly, but compared with the H + H/R + SpostC group, the ATP content of the DMOG group and the MIF20 group increased significantly (Figure 5B). Δψm, a sign of stage apoptosis was evaluated in cardiomyocytes using JC-1 staining. These results showed that the ratio of red to green fluorescence intensity in the N + H/R group was lower than in the N + CON group. But HIF-1α agonist DMOG and MIF agonist MIF20 increased the ratio of red to green fluorescence intensity and HIF-1α inhibitor YC-1 and MIF inhibitor ISO-1 blocked the effect of SPostC (Figure 5C).

## Discussion

In this study, cardiomyocytes were used to explore the potential mechanism of SPostC’s cardioprotection through HIF-1α/MIF/AMPK signaling. Previous clinical and basic research has shown that SPostC has a protective effect on myocardial I/RI (Wang et al., 2013; Shiraishi et al., 2019). Research has shown that used SPostC to treat Sprague-Dawley rats with I/RI and showed that SPostC significantly reduced the infarct size and improved cardiac function by activation of the HIF-1 pathway (Xie et al., 2014). SPostC promoted autophagosome clearance *in vitro*, reduced cell damage, and enhanced cell viability to reduce hypoxia-reoxygenation (H/R) injury in H9c2 cells (Yang et al., 2019). Here, the findings from the present study showed that SPostC exerts a protective effect for H/R injury of H9c2 cardiomyocytes by HIF-1α/MIF/AMPK signaling pathway. This cardioprotective effect is weakened or disappeared under high glucose conditions, but upregulated the expression of HIF-1α and MIF proteins, SPostC myocardial protection can be restored.

HIF-1α is an oxygen-sensitive transcription factor that enables organisms to adapt to hypoxia by transcriptional activation of up to 200 genes and is considered to be the master switch of hypoxic and ischemic signaling (Heinl-Green et al., 2005).HIF-1α regulation plays a protective role through intracellular oxygen homeostasis (Liu et al., 2020), angiogenesis (Zhou et al., 2017), embryonic cell cardiac differentiation (Gasiūnienė et al., 2019). Previous studies have found that SPostC exerts myocardial protection through upregulated HIF-1α (Yang et al., 2016). HIF-1α could upregulate the expression of MIF protein (Ma et al., 2010; Qi et al., 2014), MIF plays a cardioprotective effect by mediating AMPK phosphorylation (Hong et al., 2020). In this study, compared with the control group, in normal cultured cells, H9c2 cardiomyocytes are damaged undergoing H/R injury that showed the cell survival rate decreased, and the apoptosis rate and ROS synthesis increased. However, the expression of HIF-1α, MIF, AMPKα and p-AMPKα protein increased significantly after SPostC, accompanied by increased cell viability, decreased cell apoptosis rate, decreased ROS content, and increased ATP synthesis. It shows that SPostC could significantly resist H/R injury of cardiomyocytes. And after using HIF-1α inhibitor YC-1, the protective effect of SPostC disappears. Verify again that SPostC plays a protective role against myocardial H/R injury through the HIF-1α pathway. H9c2 cardiomyocytes cultured under high glucose have more serious cell damage after H/R injury, and SPostC cannot exert myocardial protection.

Macrophage migration inhibitory factor (MIF) is a pleiotropic inflammatory cytokine produced by immune cells and non-immune cells (Bilsborrow et al., 2019), play an important role in the inflammatory response and immune regulation (Calandra and Bucala, 1997; Burger et al., 2002). Ample evidence shows that MIF is involved in the regulation of heart function under pathological conditions, including burns, diabetes and I/RI (Pohl et al., 2016; Yu et al., 2019; Farr et al., 2020). Studies have also confirmed that MIF plays a crucial role in myocardial protection by ischemic preconditioning (Ruze et al., 2019), Ischemic preconditioning activates RISK and AMPK signal pathways through MIF, reduced myocardial infarction area and cardiomyocyte apoptosis (Dow et al., 2012; Emontzpohl et al., 2019), inhibition of ROS production (Yang et al., 2010) and inflammatory infiltration of cells, maintained endothelial cell function (Argaud et al., 2005), thereby improving cardiac dysfunction caused by myocardial I/RI (Vinten-Johansen et al., 2007). Studies have also shown that MIF inhibits c-Jun N-terminal kinase (JNK) mediated apoptosis (Qi et al., 2009), and provides myocardial protection during myocardial I/RI through its antioxidant capacity.

In this study, the MIF inhibitor IOS-1 was used in normal cultured cells. It was found that after inhibiting the expression of MIF, the protective effect of SPostC disappeared. AMPK is used as a sensor of cell energy metabolism. The study found that the inhibition of MIF caused the decrease of p-AMPKα, accompanied by the decrease of mitochondrial membrane potential and ATP synthesis. However, the results of the SPostC group showed that the ATP content was increased and the mitochondrial membrane potential was restored. Therefore, it is confirmed that SPostC exerts a cardioprotective effect through the MIF/AMPK signal axis, thereby promoting cell energy metabolism. The research results also show that the expression of HIF-1α, MIF, AMPKα and p-AMPKα is correlated in the process of SPostC. Therefore, healthy cardiomyocytes undergo H/R injury, and MIF is another target for SPostC to exert its cardioprotective effect. It shows that on healthy cardiomyocytes, HIF-1α/MIF/AMPK signal transduction is the key link of SPostC against H/R injury of cardiomyocytes.

ATP production in adult myocardium is mainly (more than 95% part) provided by mitochondrial oxidative phosphorylation (Ashrafian et al., 2007). However, myocardial I/RI requires anaerobic glycolysis to obtain energy. Studies have shown that HIF-1α plays an important role in anaerobic glycolysis (Lu et al., 2002). HIF-1α regulates almost all enzymes that cause glucose breakdown during glycolysis by activating lactate dehydrogenase A (LDH-A) and lactate transporter 4 (MCT4) (Semenza, 2010). HIF-1α also inhibits in many ways Mitochondrial respiratory chain function (Selak et al., 2005; Kim et al., 2006; Papandreou et al., 2006; Zhang et al., 2007). Experiments on cells cultured under hyperglycemia and hypoxia have shown increased degradation of HIF-1α protein (Catrina et al., 2004; Ramalho et al., 2017), consistent with the results of this study. The results show that diabetes not only causes hypoxia but also affects HIF-1α signaling. Therefore, verified again the previous research results of our team (Yu et al., 2017), the reason for the weakened protective effect of SPostC in the diabetic state is due to the damage of the HIF-1α pathway.

Therefore, here cardiomyocytes were cultured with high glucose using the HIF-1α agonist DMOG, and MIF agonist MIF recombinant protein (MIF20) respectively found that the cardioprotective effect of SPostC is restored by up-regulating the expression of HIF-1α or MIF protein. It was found that cell H/R injury was improved which was manifested by increased ROS production, decreased ATP synthesis, and decreased mitochondrial membrane potential. At the same time, it was found that the protein levels of p-AMPKα increased significantly. It shows that myocardial protection of SPostC was reversed by up-regulating the expression of HIF-1α or MIF protein. The mechanism of the SPostC myocardial protective is mainly due to the improvement of the phosphorylation level of downstream AMPK, thereby improving the energy metabolism of the myocardium. The results show that regulating HIF-1α/MIF/AMPK signaling pathway could restore the protective effect of SPostC on myocardial culture in high glucose.

There are still some limitations in this study. We simply used H9c2 cardiomyocyte cell. Further experiments are to verify the role of HIF-1α/MIF/AMPK signaling in SPostC through *in vitro* and vivo. Drugs were used to inhibit or agonist HIF-1α and MIF in this study instead of silencing or knockout techniques and adenovirus transfection.

In summary, the HIF-1α/MIF/AMPK signaling pathway may be the internal mechanism of SPostC’s protective effect. The HIF-1α/MIF/AMPK signal axis may be the key link for SPostC to combat H/R injury of cardiomyocytes under high glucose conditions. Therefore, a large number of related studies are needed to provide a theoretical basis for the application of SPostC in clinical anesthesia to exert myocardial protection.

## Data Availability

The original contributions presented in the study are publicly available. This data can be found here: https://pan.baidu.com/s/10isjozjKkrWbDyPBrjqsSA accession number u8j0.
